# The long noncoding RNA, treRNA, decreases DNA damage and is associated with poor response to chemotherapy in chronic lymphocytic leukemia

**DOI:** 10.18632/oncotarget.15401

**Published:** 2017-02-16

**Authors:** Cecelia R. Miller, Amy S. Ruppert, Sydney Fobare, Timothy L. Chen, Chaomei Liu, Amy Lehman, James S. Blachly, Xiaoli Zhang, David M. Lucas, Michael R. Grever, Martin S. Tallman, Ian W. Flinn, Laura Z. Rassenti, Thomas J. Kipps, Deepa Sampath, Kevin R. Coombes, Erin K. Hertlein

**Affiliations:** ^1^ Department of Internal Medicine, Division of Hematology, Comprehensive Cancer Center at The Ohio State University, Columbus, OH, USA; ^2^ Hendrix College, Conway, AR, USA; ^3^ Department of Experimental Therapeutics, MD Anderson Cancer Center at University of Texas, Houston, TX, USA; ^4^ Center for Biostatistics, The Ohio State University, Columbus, OH, USA; ^5^ Memorial Sloan Kettering Cancer Center, New York, NY, USA; ^6^ Sarah Cannon Research Institute and Tennessee Oncology, Nashville, TN, USA; ^7^ Department of Medicine, Moores Cancer Center at University of California at San Diego, La Jolla CA, USA; ^8^ Department of Biomedical Informatics, The Ohio State University, Columbus, OH, USA; ^9^ The Chronic Lymphocytic Leukemia (CLL) Research Consortium, La Jolla, CA, USA

**Keywords:** lncRNA, treRNA, chronic lymphocytic leukemia, DNA damage, prognostic factor

## Abstract

The study of long noncoding RNAs (lncRNAs) is an emerging area of cancer research, in part due to their ability to serve as disease biomarkers. However, few studies have investigated lncRNAs in chronic lymphocytic leukemia (CLL). We have identified one particular lncRNA, treRNA, which is overexpressed in CLL B-cells. We measured transcript expression in 144 CLL patient samples and separated samples into high or low expression of treRNA relative to the overall median. We found that high expression of treRNA is significantly associated with shorter time to treatment. High treRNA also correlates with poor prognostic indicators such as unmutated *IGHV* and high ZAP70 protein expression. We validated these initial findings in samples collected in a clinical trial comparing the nucleoside analog fludarabine alone or in combination with the alkylating agent cyclophosphamide in untreated CLL samples collected prior to starting therapy (E2997). High expression of treRNA was independently prognostic for shorter progression free survival in patients receiving fludarabine plus cyclophosphamide. Given these results, in order to study the role of treRNA in DNA damage response we generated a model cell line system where treRNA was over-expressed in the human B-CLL cell line OSU-CLL. Relative to the vector control line, there was less cell death in OSU-CLL over-expressing treRNA after exposure to fludarabine and mafosfamide, due in part to a reduction in DNA damage. Therefore, we suggest that treRNA is a novel biomarker in CLL associated with aggressive disease and poor response to chemotherapy through enhanced protection against cytotoxic mediated DNA damage.

## INTRODUCTION

Chronic lymphocytic leukemia (CLL) is a B-cell malignancy with a heterogeneous clinical course. While some patients have indolent disease that may not require therapy for years, others progress quite rapidly. Presently, biomarkers such as unmutated *IGHV*, ZAP70 expression, and cytogenetic deletion of 17p or 11q have been identified as independent markers for aggressive disease [1–4]. However, these markers are insufficient to demarcate all aggressive cases. Identifying additional molecular markers that predict for aggressive disease or that defines a potentially different biology remains of considerable interest, particularly those which can predict response to treatment.

Gene expression can be a useful prognostic indicator in CLL, particularly the expression of noncoding RNAs such as microRNAs (miRs). Several miRs have been shown to play critical biological roles in CLL, particularly in cell signaling. For example, mir-155 expression enhances sensitivity to B-cell receptor (BCR) ligation [5], and high expression of miR-155 is associated with shorter overall survival and progression free survival with chemo-immunotherapy [5, 6]. Conversely, miR-150 expression has been shown to decrease the intensity of BCR signaling [7]. Calin et. al has described a 9 miR signature associated with decreased time to treatment (TTT) in CLL that includes mirR-181a (high), miR-155 (high), miR-146 (high), and miR-29c (low) [8]. Negative regulators of the anti-apoptotic protein BCL2, miR-15 and miR-16, are often chromosomally deleted or downregulated in CLL [9, 10]. While the expression of miRs has been clearly demonstrated as important in CLL, the contribution of other noncoding RNAs has not been well established in this disease. Recently, it has been shown that long noncoding RNAs (lncRNAs; defined as noncoding transcripts greater than 200 nucleotides in length) are able to regulate gene expression and are associated with diverse biological processes [11, 12]. The deregulated expression of lncRNAs such as HOTAIR, MALAT1, and SChLAP1 have been associated with disease progression in multiple types of cancer [13–18]. Specifically in CLL, differential expression of lncRNAs has been identified across molecular subgroups as well as compared to normal B-cells [19, 20]. Even so, few functional studies have been done to determine the role of lncRNAs in CLL disease mechanism although they likely play a significant part in this process. Some examples include linc-p21 and NEAT1, which have been shown to be induced by DNA damage in a p53 dependent mechanism in CLL cells [21]. In addition, circulating levels of linc-p21 in plasma from CLL patients is lower than healthy donor controls [22].

In the current study, we explored the differential expression of lncRNAs in CLL and identified treRNA (*TRERNA1*), as overexpressed in CLL and associated with poor prognosis. TreRNA has been described to have enhancer-like function as well as translational regulatory functions, and is overexpressed in breast cancer lymph-node metastases and colon cancer [23, 24]. In CLL, we found high expression of treRNA was associated with poor response to chemotherapy independent of other variables, and suggest that treRNA may serve as a valuable prognostic factor in this disease. Finally, using a CLL cell line model we show that over-expression of treRNA results in decreased DNA damage caused by exposure to chemotherapeutic agents, likely contributing to the impaired response and decreased clinical benefit observed in patients receiving these agents.

## RESULTS

### lncRNAs are differentially expressed in CLL

**Figure 1:**
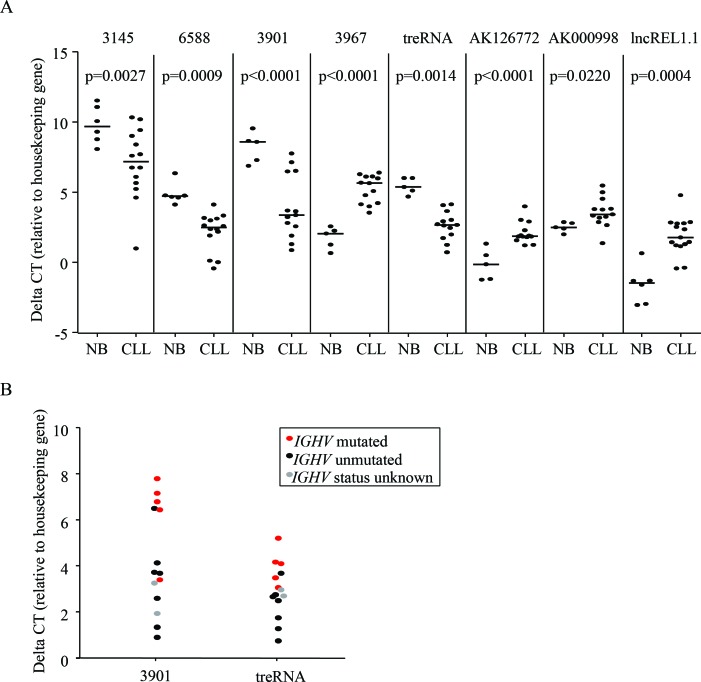
LncRNAs are aberrantly expressed in CLL **a**. Relative expression of lncRNAs in primary CLL cells compared to normal B cells measured by qRT-PCR. P-values were determined by linear mixed effects models. **b**. Relative expression of treRNA and ENST00000413901 in *IGHV* unmutated (black) compared to *IGHV* mutated (red) primary CLL cells. Gray dots are samples with unknown mutational status. DeltaCT = (lncRNA CT - housekeeping gene CT). P-values were determined by two-sided *t*-tests; *p* = 0.0395 for ENST00000413901 and *p* = 0.0125 for treRNA.

We first sought to explore if lncRNAs are differentially expressed in CLL by comparing pooled RNA isolated from CLL B-cells to pooled RNA from healthy donor peripheral blood B-cells, with and without stimulation with CD40 ligand. This was done using the ArrayStar human lncRNA microarray, a platform that analyzes over 30,000 lncRNA transcripts in addition to 30,000 coding transcripts. 2418 probes, including both mRNA and lncRNA, were significantly different between CLL and normal B-cells at a FDR = 10% (Supplementary Figure 1). We chose 14 transcripts annotated as lncRNA for further validation, selected based on the degree of differential expression and potential relevance to cancer based on previous publications. We analyzed these targets in an additional 14 CLL samples and 5 normal B-cell samples (independent of those used in the microarray), and were able to validate 8 lncRNAs as having significantly different expression when comparing CLL samples to normal B-cell samples (Figure 1A). In addition, two of these lncRNAs, treRNA and ENST00000413901, exhibited significantly higher expression in the unmutated *IGHV* samples compared to mutated (Figure 1B; *p* = 0.0395 for treRNA and *p* = 0.0125 for ENST00000413901). Based on this potential relevance to aggressive CLL disease, we further investigated the prognostic significance of these two lncRNAs in a large well-characterized cohort of patient samples.

### High expression of treRNA is associated with shorter time to treatment

**Figure 2:**
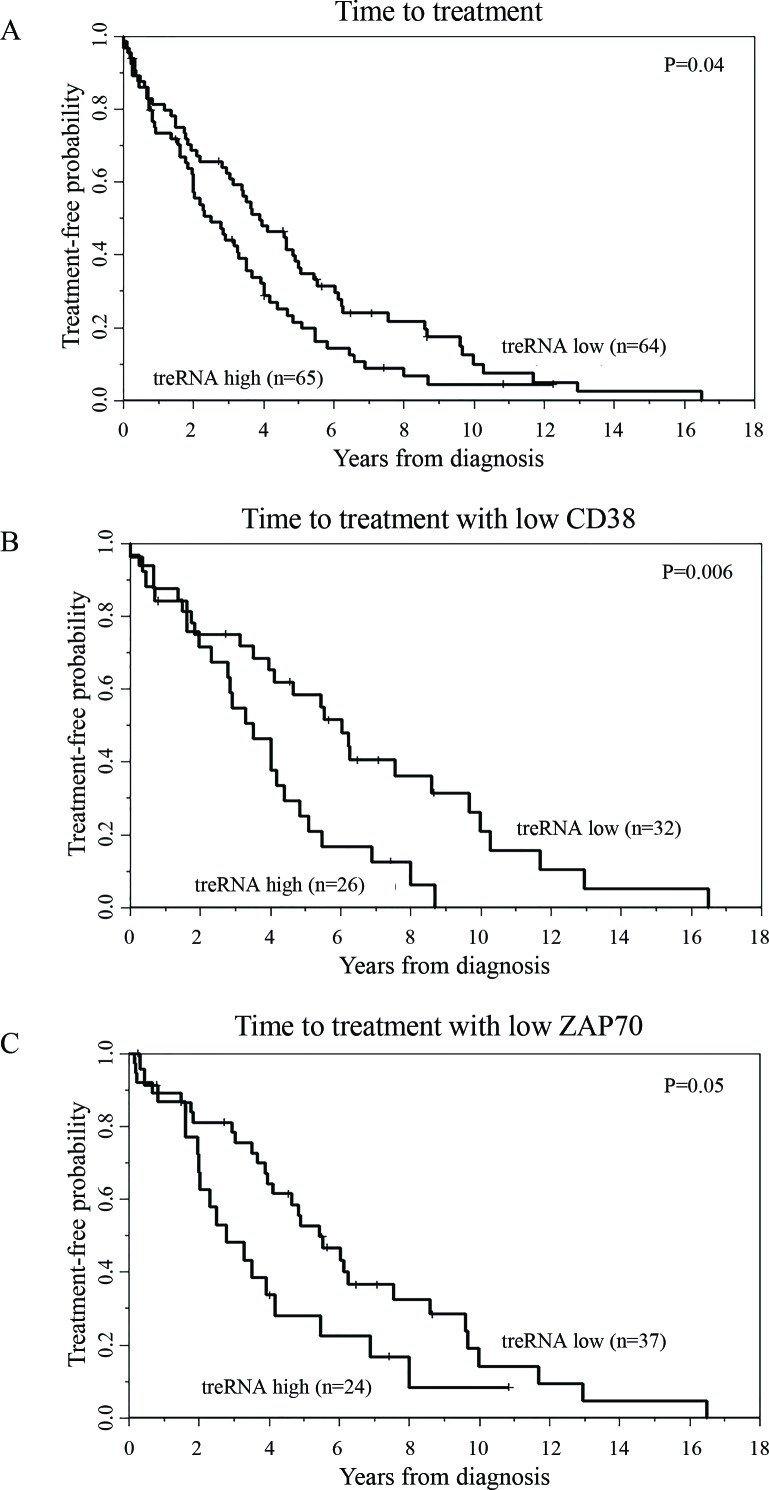
TreRNA is associated with time to treatment in CLL Time to treatment in the **a**. CRC patient set **b**. CD38 negative patients, and **c**. ZAP70 protein expression negative patients. *P*-values were determined by the log-rank test.

The initial set of CLL samples used to assess prognostic significance of treRNA and ENST00000413901 (abbreviated 3901) were obtained from 144 previously untreated asymptomatic CLL patients enrolled in the CRC registry. In this particular patient set, both ZAP70 protein expression and methylation status (a surrogate marker for Zap70 expression) has been previously described [4]. Expression of treRNA and ENST00000413901 were measured using qRT-PCR and normalized to the housekeeping gene TBP. We separated patient samples into high or low expression of treRNA and ENST00000413901 based on the median expression value within the set. We found that high expression of treRNA associated with poor prognostic indicators: low ( < 20%) *ZAP70* methylation (*p* < 0.001), high ( > 20%) ZAP70 protein expression (*p* = 0.003), and unmutated *IGHV* (*p* < 0.0001) (Table 1). High expression of treRNA also associated with shorter TTT across the entire dataset (*p* = 0.04, Figure 2A). Interestingly, high expression of treRNA identified patients with shorter TTT within two different favorable prognostic subgroups: low CD38 expression (*p*= 0.006, Figure 2B) and low ZAP70 protein expression (*p* = 0.05, Figure 2C). Although lncRNA ENST00000413901 was also associated with poor prognostic indicators low *ZAP70* methylation, high ZAP70 protein expression, and unmutated *IGHV* (data not shown), it was not as strongly associated with TTT. Of these two lncRNAs, treRNA exhibits better correlation with CLL disease markers, and the transcript has been previously well characterized. We therefore focused our remaining correlative and functional studies on treRNA.

**Table 1:** TreRNA is associated with aggressive disease markers in the CRC patient set

Variable	Low treRNA (*n*= 72)	High treRNA (*n*= 72)	*P*-value
Median Age, yrs. (Range)	53 (32-78)	52 (26-82)	0.33
Female, Num. (%)	18 (25)	26 (36)	0.21
ZAP70 Methylation, Num. (%) Low High (> 20%)	44 (61) 28 (39)	63 (88) 9 (13)	**<0.001**
ZAP70 Protein Expression, Num. (%) Negative Positive (>20%)	45 (63) 27 (38)	26 (36) 46 (64)	**0.003**
CD38 Expression, Num. (%) Negative Positive (>20%)	39 (54) 33 (46)	31 (43) 41 (57)	0.24
IGHV, Num. (%) Mutated Unmutated (>98%)	29 (40) 43 (60)	8 (11) 64 (89)	**<0.001**

Associations were tested using the Wilcoxon rank sum and Fisher's exact tests. Abbreviations: yrs, years; num, number; del, deletion.

### High expression of treRNA independently predicts shorter progression free survival in patients receiving chemotherapy

**Figure 3:**
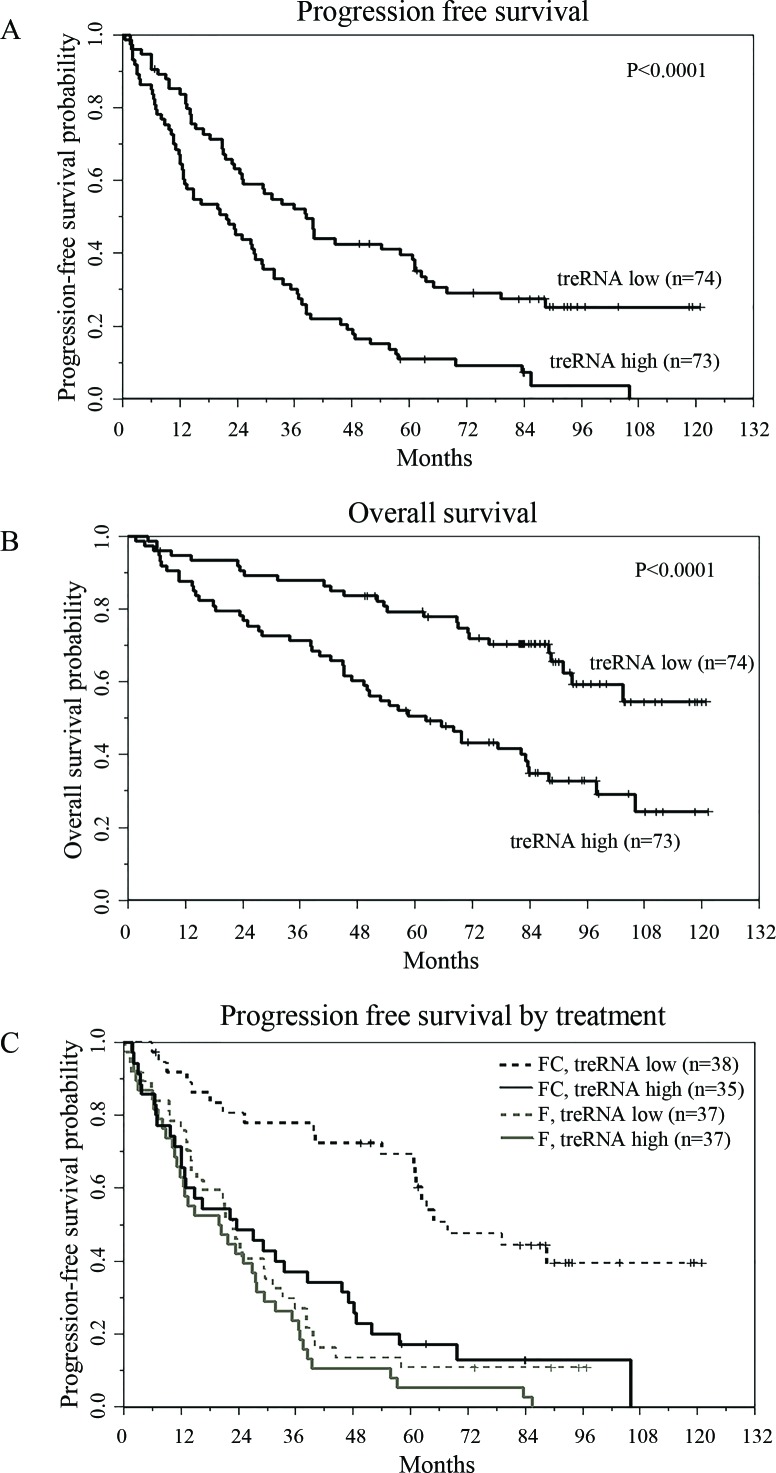
TreRNA is associated with progression-free survival in CLL **a**. Progression-free survival, **b**. overall survival, and **c**. progression-free survival by treatment arm and by treRNA group. P-values were determined by the log-rank test.

While interrogating the clinical significance of treRNA we discovered that CLL cells contain a transcript in the nuclear RNA fraction that retains the intron between the two coding exons, likely due to insufficient splicing (Supplementary Figure 2A & 2B). Therefore we investigated the prognostic significance of both spliced and retained intron treRNA (ri-treRNA) in a second, independent dataset. For this we used 147 (pre-treatment) samples from symptomatic, previously untreated patients enrolled in the Eastern Cooperative Oncology Group E2997 (E2997) phase III clinical trial comparing the nucleoside analog fludarabine to the combination of fludarabine plus the alkylating agent cyclophosphamide [25]. In this setting, we confirmed the association between high expression of treRNA and unmutated *IGHV* (*p* = 0.0029), and identified an additional association with cytogenetic markers; namely high treRNA expression occurred more frequently in patients with deletion 17p, and less frequently with trisomy 12 (*p* = 0.018) (Table 2). High treRNA was also associated with shorter PFS (*p* < 0.0001; Figure 3A) and overall survival (OS) (*p* < 0.0001; Figure 3B). Even though ri-treRNA expression correlates with treRNA expression (Supplementary Figure 3A), these associations were not significant for the unspliced transcript (Supplementary Figure 3B). This could be related to the fact that patients express considerably lower levels of ri-treRNA compared to spliced treRNA and have a smaller dynamic range of expression. We next included treRNA expression in a multivariable model which has been previously described for this dataset [26, 27]. TreRNA expression did not add prognostic information for OS in the multivariable model once accounting for treatment arm, age, and molecular group. However, the impact of treRNA on PFS was significantly different depending on whether patients received fludarabine plus cyclophosphamide or fludarabine alone (*p* = 0.01) (Figure 3C). In a model accounting for the effect modification of treatment arm on the relationship between treRNA and PFS and adjusting for age, sex, Rai stage, and molecular group, high treRNA expression was strongly associated with a higher risk of progression or death in the group who received fludarabine plus cyclophosphamide (HR 3.14, 95% CI 1.61-6.14, *p* = 0.0008). For those who received fludarabine alone, PFS was short irrespective of treRNA expression (HR 1.12, 95% CI 0.62-2.02, *p* = 0.70).

**Table 2:** TreRNA is associated with aggressive disease markers in the ECOG 2997 patient set

Variable	Low treRNA (*n* = 74)	High treRNA (*n* = 73)	*P*-value
Treatment Arm, Num. (%) Fludarabine (F) F+Cyclophosphamide (C)	37 (50) 37 (50)	38 (52) 35 (48)	0.87
Median Age, yrs. (Range)	62 (33-83)	60 (42-78)	0.34
Female, Num. (%)	20 (27)	22 (30)	0.72
Rai Stage II/III/IV, Num. (%)	51 (69)	56 (77)	0.35
ZAP70 Methylation, Num. (%) Low High (>20%) Unknown	32 (70) 14 (30) 28	45 (79) 12 (21) 16	0.36
IGHV, Num. (%) Mutated Unmutated (>98%) Unknown	29 (49) 30 (51) 15	16 (23) 53 (77) 4	**0.0029**
Dohner Classification, Num. (%) del(17p) del(11q) +12 del(6q) Normal del(13q) Unknown	2 (3) 8 (11) 25 (34) 1 (2) 13 (18) 24 (33) 1	11 (15) 11 (15) 12 (17) 4 (6) 11 (15) 22 (31) 2	**0.018**
Molecular Group, Num. (%) del(17p) del(11q) +12/Notch Mutated IGHV Unmutated Other Unknown	2 (3) 8 (13) 11 (18) 15 (25) 25 (41) 13	11 (16) 11 (16) 4 (6) 32 (48) 9 (13) 6	**<0.0001**

Associations were tested using the Wilcoxon rank sum and Fisher's exact tests. Abbreviations: yrs, years; num, number; del, deletion.

### The presence of treRNA results in less DNA damage with fludarabine and mafosfamide

**Figure 4:**
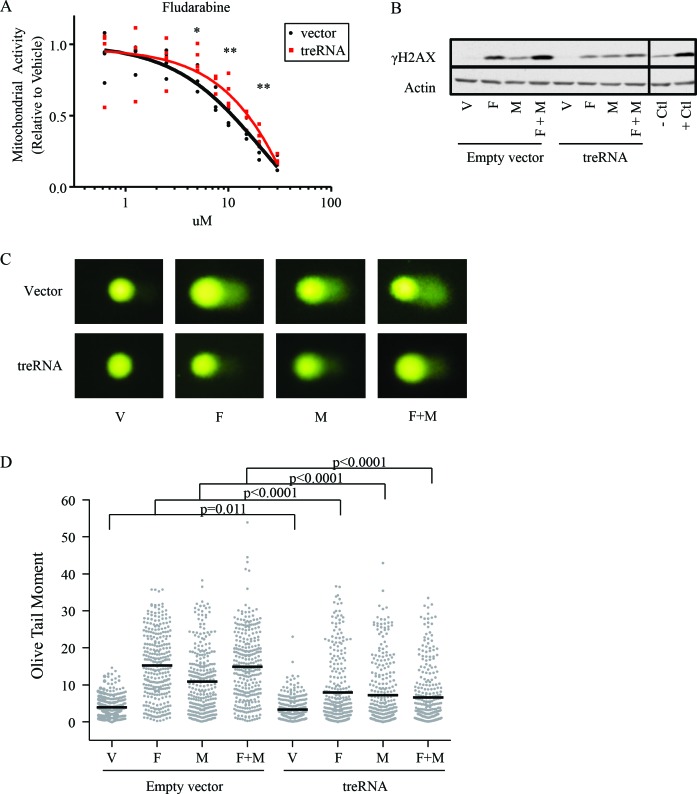
TreRNA expression impairs DNA damage following treatment with fludarabine and mafosfamide **a**. Viability, measured by MTS, of OSU-CLL treRNA and OSU-CLL empty vector after 48 hours of fludarabine treatment. Data are shown with individual replicates and best fit lines. P-values were determined by linear mixed effects models. *, *p* = 0.002; **, *p* < 0.001. **b**. Representative immunoblot of γH2AX induction in OSU-CLL-treRNA *versus* empty vector cell lines following 24 hour treatment with fludarabine (F, 10uM), mafosfamide (M, 2.36uM) or a combination of fludarabine plus mafosfamide (F+M). Actin probed as a loading control. Image cropped to show relevant cell lines and controls. **c**. Representative images from comet assay after one hour of drugging with F, M, or F+M in OSU-CLL empty vector and OSU-CLL treRNA. **d**. Quantitation of comet assay using olive tail moment for 200 cells per each condition. P-values were determined by linear mixed effects models.

The inferior progression-free survival with fludarabine plus cyclophosphamide in patients with high treRNA expression suggests a role for treRNA in mediating DNA damage response; therefore we established a stable retroviral system to further study this observation *in vitro*. We used the CLL cell line (OSU-CLL; previously established in our lab [28]) to exogenously express treRNA, as well as a control line transduced with the empty viral vector. Expression of treRNA did not alter baseline viability, proliferation, or migration (Supplementary Figure 4). When treated with chemotherapeutic agents, the treRNA cell line exhibited increased viability compared to the vector control when drugged with fludarabine at 5, 10, and 20uM; this protective effect diminished at 30uM (Figure 4A). Expression of treRNA was less protective to mafosfamide, a significant difference in viability between the treRNA and control cell line was seen only at the highest concentration of drug (10uM) (Supplementary Figure 5A). In OSU-CLL expressing treRNA, we observed a trend towards less induction of the DNA damage indicator, γH2AX, when exposed to fludarabine or fludarabine plus mafosfamide, but not mafosfamide alone (Figure 4B), although this did not reach statistical significance (Supplementary Figure 5B). Therefore we verified the difference in DNA damage using the more quantifiable comet assay. Following one hour incubation with fludarabine, mafosfamide, or the combination, DNA damage was markedly less induced in all drugging conditions in OSU-CLL treRNA compared to empty vector, indicated by the shorter comet tails (Figure 4C). Quantitation of the damage for 300 individual cells per condition (measured by olive tail moment) verified significantly less induction in the treRNA expressing cell line (*p* < 0.0001; Figure 4D). These results indicate that the presence of treRNA results in decreased DNA damage in cells exposed to chemotherapeutic agents.

## DISCUSSION

Here we characterized the differential expression of lncRNA in CLL cells as compared to normal blood B-cells. While validating differentially expressed lncRNA identified by microarray, we narrowed our interest to two lncRNAs, treRNA and ENST00000413901, due to their variable expression in CLL patients and association with *IGHV* mutational status. Of these two, only treRNA was significantly associated with TTT in the initial prognostic dataset. While we did not pursue further studies with ENST00000413901, the clear separation of patients who highly express this lncRNA in contrast to patients with low to undetectable levels suggests that this lncRNA may reflect biologically distinct subgroups although such expression did not vary with clinical outcome.

Our initial dataset indicated that patients with high expression of treRNA are enriched for aggressive disease markers and required treatment earlier than those with lower expression. These findings were supported in a second, independent dataset that confirmed the association between treRNA and the aggressive *IGHV* unmutated disease, and also identified that treRNA expression is associated with shorter PFS and OS. Interestingly, we found low expression of treRNA expression was an independent prognostic factor for improved PFS in patients receiving fludarabine plus cyclophosphamide. TreRNA expression was not associated with PFS in patients receiving fludarabine alone, however, this arm of the study overall did very poorly. It would it be interesting to see if the prognostic significance of treRNA is specifically associated with therapy involving these DNA damaging agents and if this association with PFS will persist for patients receiving fludarabine plus cyclophosphamide in combination with rituximab, as this is a standard treatment strategy in CLL [29, 30]. Given that treRNA has been reported as expressed in breast cancer lymph-node metastases as well as colorectal cancer [24], and that chemotherapeutic agents are also used for treatment of these types of cancer, it would also be of interest to explore if treRNA expression is predictive of chemotherapeutic response in these solid tumors.

The association with PFS of the combination treatment of fludarabine plus cyclophosphamide suggests that treRNA may play a role in response to DNA damaging agents, prompting further exploration of this. *In vitro* studies using the OSU-CLL cell line expressing treRNA showed increased viability and a slight trend towards less induction of the DNA damage marker γH2AX. We validated this trend using the comet assay, which more readily quantifies DNA repair, and found that OSU-CLL expressing treRNA had markedly reduced induction of DNA damage after one hour exposure to fludarabine, mafosfamide, and the combination compared to OSU-CLL empty vector. Interestingly, these protective effects were more pronounced with fludarabine exposure compared to mafosfamide. These results suggest that treRNA may be directly involved in decreasing DNA damage after exposure to chemotherapeutic agents and provides a basis for why this marker is independently associated with PFS in patients who receive these agents. However, the cell line model may be unable to fully recapitulate what is happening in the patient cells due to inherent differences between cell lines and primary samples, the lack of microenvironment interactions in the *in vitro* setting, and this model does not address the role of treRNA with prolonged exposure to chemotherapeutic agents.

TreRNA has been reported to affect both transcription and translation; however, the targets of treRNA may be cell type specific [23, 24]. In normal B-cells treRNA was low to undetectable; however, in CLL patients samples we found a wide range of expression. High treRNA expression in CLL correlated with poor prognostic markers suggesting that treRNA expression is part of an aggressive B-cell leukemia phenotype. However, the factors that drive the expression of treRNA, and functional effects of treRNA outside of DNA damage response in CLL, remain to be determined.

LncRNAs have been reported to function as regulators of gene expression and can directly contribute to aggressive cancer phenotypes. Our results suggest lncRNAs are significantly deregulated in CLL compared to normal B-cells, and can be used as prognostic indicators in this disease. While our results elucidate the role for a particular lncRNA (treRNA) in the response to DNA damage, it is likely that other lncRNA profiles will be predictive for other types of therapeutics used in CLL, such as B-cell receptor antagonists. Therefore further investigation into the function of aberrantly expressed lncRNA may help us further understand CLL pathogenesis, and provide important insight into response to therapy.

## MATERIALS AND METHODS

### Patient samples and cell culture conditions

Peripheral blood was obtained from CLL patients with written informed consent in accordance with the Declaration of Helsinki and under protocols approved by the Institutional Review Board of the Ohio State University. CLL cells and normal B-cells were isolated using ficoll density gradient centrifugation (Ficoll-Plaque Plus; Amersham Biosciences) and enriched for B-cells using the Rosette-Sep negative selection kit (StemCell Technologies) according to manufacturer protocol. Cryopreserved cells used in the two prognostic datasets were obtained from the CLL Research Consortium (CRC) tissue bank or from the ECOG-2997 clinical trial. Cells were thawed in RPMI 1640 media then washed in PBS to obtain cell pellets.

The OSU-CLL cell line was grown in RPMI 1640 media supplemented with 10% fetal bovine serum, 2mM L-glutamine (Invitrogen), 100U/mL penicillin, and 100ug/mL streptomycin (Sigma). For DNA damage experiments, 2×10^6^ cells were incubated with vehicle (DMSO), 10uM fludarabine (Sigma), 2.36uM mafosfamide (Santa Cruz), or 10uM fludarabine plus 2.36uM mafosfamide. All conditions were given equivalent volumes of DMSO.

### Microarray analysis

Using ArrayStar Human LncRNA Array v2.0, two pools of RNA from CLL patient peripheral blood B-cells (*n* = 5 per pool) with mixed clinical and molecular histories, were compared to a pool of healthy donor B-cells (*n* = 6) isolated from Red Cross leukopaks. The normal B-cells and CLL B-cells were either unstimulated or stimulated with CD40 ligand (PeproTech). As an exploratory experiment to identify potential differentially expressed genes for further validation, the pooled CLL samples and pooled normal B-cells samples (unstimulated and stimulated) were treated as independent samples in the statistical analysis. All analyses were performed in version 3.1.0 of the R Statistical Programming Environment. Data processing was performed with version 3.20.1 of the limma package. Background correction was performed using the “normal-exponential” model, and then quantile normalization applied. Probe-by-probe smoothed *t*-tests were used to identify genes differentially expressed between CLL cells and normal B-cells. In order to account for multiple testing, a beta-uniform-mixture (BUM) model was used on the p-values to estimate the false discovery rate (FDR). Microarray results are accessible at GEO entry # GSE87575.

### Quantitative reverse transcription PCR (qRT-PCR)

RNA was extracted by phenol chloroform isolation using TRIzol reagent (Invitrogen) then purified using the MirVANA kit total RNA isolation procedure (Ambion). Following isolation RNA was treated with Turbo DNase (Ambion). cDNA was prepared with SuperScript First-Strand Synthesis System (Invitrogen). qRT-PCR to validate the microarray was performed using SybrGreen master mix (Applied Biosystems). Prognostic datasets were run using TaqMan master mix (Applied Biosystems). Detection was performed using an ABI Prism 7700 detection system (Applied Biosystems). LncRNA expression was normalized to internal control genes TATA-box binding protein (TBP) or Glyceraldehyde 3-phosphate dehydrogenase (GAPDH). Custom-designed primers were used for all SybrGreen reactions. Custom TaqMan primers were used for treRNA and ENST00000413901 TaqMan reactions, with TaqMan TBP primer #4326322E (ThermoFisher) used as the internal control. Custom designed primer sequences are provided in supplemental information (Supplementary Table 1). DeltaCT was determined by subtracting the CT value of the housekeeping gene from the CT value of the lncRNA.

### Nuclear and cytoplasmic RNA extraction

Nuclear and cytoplasmic fractions were isolated using the NE-PER kit (Thermo Scientific). RNA was then collected using the miRVANA kit (Ambion) using the manufacturer protocol for cultured cells followed by total RNA extraction. Following isolation RNA was treated with Turbo DNase (Ambion). Standard PCR was performed for treRNA, U2, and S14 as previously described by Gummireddy et al [24].

### Retrovirus vectors and generation of treRNA expressing cell lines

A 589bp PCR product encoding the spliced transcript of treRNA was cloned into the EcoRI/Not I sites of pRetro-tight-puro (Clontech). pRetro-tight-puro vector without insert was used as a control. Retrovirus was produced by co-transfecting the plasmid DNA of interest and ecotropic-helper plasmids (pVSV and pGPZ) into the 293T cell line using calcium phosphate precipitation. Doxycycline inducible Tet activator OSU-CLL cell line (pRetrox-Tet-on) was established according to the manufacturer's protocol (Clontech). OSU-CLL-pTet-on cells were then infected with retrovirus by culturing for 10 hours in the conditioned media with 8 μg/mL of polybrene. Cells were then washed to eliminate virus and polybrene and incubated in complete media for 48 h before selection with 1μg/mL of puromycin and 500ug/mL G418.

### Viability

Cytotoxicity was measured using 3-(4,5-dimethylthiazol-2-yl)-5-(3-carboxymethoxyphenyl)-2-(4-sulfophenyl)-2H-tetrazolium (MTS) assays. Doxycycline induced OSU-CLL treRNA and empty vector cells were plated at 100,000 cells per well in a 96 well plate and incubated 48 hours in fludarabine, mafosfamide, or vehicle. MTS reagent was then added and spectrophotometric readings were taken 4 hours later. Apoptosis was assessed by staining with Annexin V- FITC and propidium iodide (PI). Data were collected on a Beckman Coulter FC500 flow cytometer then analyzed using Kaluza software.

### Migration

Cells were suspended in RPMI at 5×10^6^cells/mL, and 100 μl was placed in the upper chamber of a 24-transwell plate with a 5μm filter. Chambers were placed into wells containing media containing no chemokine (control), recombinant human CXCL12 (200ng/mL, Millipore) or CXCL13 (1000ng/mL, R&D systems). Migration was permitted for 3 hours, and cells in the lower chamber were collected and counted for 20 seconds on high speed on a Beckman Coulter FC500 flow cytometer. A 1/20 dilution of input cells was also determined.

### Proliferation

Proliferation was measured using the Click-iT Plus EdU Alexa Fluor 488 Flow Cytometry Assay Kit (Invitrogen) following the manufacturer protocol. Cells were incubated with 10uM EdU for 2 hours before staining.

### Immunoblot

Whole cell lysates were prepared by lysing PBS-washed OSU-CLL cell pellets in cold lysis buffer containing phosphatase inhibitor cocktail 1 and 2, protease inhibitor cocktail P8340 and 1mM phenylmethylsulfonyl fluoride (all from Sigma). Protein was quantified by the BCA method (Pierce). Protein (25ug/lane) was separated on 12% polyacrylamide gels and transferred onto nitrocellulose. After antibody incubations, proteins were detected with chemiluminescent substrate (Advansta) and quantified using a ChemiDoc system with Quantity One software (Bio-Rad Laboratories). The following antibodies were used for detection, anti-ACTB (Santa Cruz Biotechnologies) and anti-γH2AX (Abcam).

### Single cell gel electrophoresis assay (SCGE, also known as comet assay)

Comet assay was performed using reagents from the Trevigen Comet Assay kit. Following a one hour drugging of OSU-CLL empty vector and treRNA, samples were washed with ice cold PBS then combined with comet agarose at a 1:10 ratio. Samples were adhered for 30 minutes at 4°C then immersed in lysis buffer for 60 minutes at 4°C. Samples were then submerged in alkaline unwinding buffer for 30 minutes at 4°C. Samples were electrophoresed in alkaline running buffer, fixed with 70% ethanol, and then stained with SYBR gold (Fisher). Olive tail moment was quantified using Komet imaging software (Andor technologies) for 100 cells per each condition in three experimental replicates.

### Statistical analysis

Linear mixed effects models were applied to the delta CT values (relative to control: GAPDH or TBP), allowing for dependencies among observations from the same sample. From the models, which contained the interaction between target and cell type, differences in delta CT values between CLL and normal B cells for each target were estimated, with 95% CI, and then converted to fold changes. To control the overall Type I error at 0.05, Holm's step-down procedure was used to adjust the p-values from each of the individual target comparisons (Figure 1).

Linear mixed effects models were also used were used for statistical analysis under each treatment condition for experiments involving viability (MTS; Figure 4a), western blot quantitation (log transformed, Supplementary Figure 5b), and olive tail moment (Figure 4d). Analyses were performed using SAS/STAT software, Version 9.4 of the SAS System for Windows (SAS Institute Inc.).

Clinical endpoints were defined as follows: Time to treatment (TTT) was measured from the date of diagnosis until the date of first treatment, censoring patients who had not started treatment at last follow-up. PFS was defined as the time from randomization until documented disease progression or death without progression, censoring patients alive and progression-free at the date of last reported contact, and OS was defined as the time from randomization until date of death, censoring patients alive at last contact date. Associations between lncRNAs and TTT, PFS, or OS were initially explored using Kaplan-Meier plots and differences between low and high expression groups were evaluated with the log-rank test. Multivariable models were fit using Cox proportional hazards models. Associations between treRNA with other clinical and molecular features were tested using the Wilcoxon rank sum or Fisher's exact tests for continuous and categorical variables, respectively. All tests were two-sided and statistical significance was declared for *p* < 0.05.

## SUPPLEMENTARY MATERIALS FIGURES AND TABLES


