# Supplementary material for: The long noncoding RNA, treRNA, decreases DNA damage and is associated with poor response to chemotherapy in chronic lymphocytic leukemia
Source: Oncotarget. 2017 Feb 16;8(16):25942–54. doi: 10.18632/oncotarget.15401 (PMC5432228; doi:10.18632/oncotarget.15401)
Supplement: Supplementary file 1 [file oncotarget-08-25942-s001.pdf]

The long noncoding RNA, treRNA, decreases DNA damage and is associated with poor response to chemotherapy in chronic lymphocytic leukemia

Supplementary Material

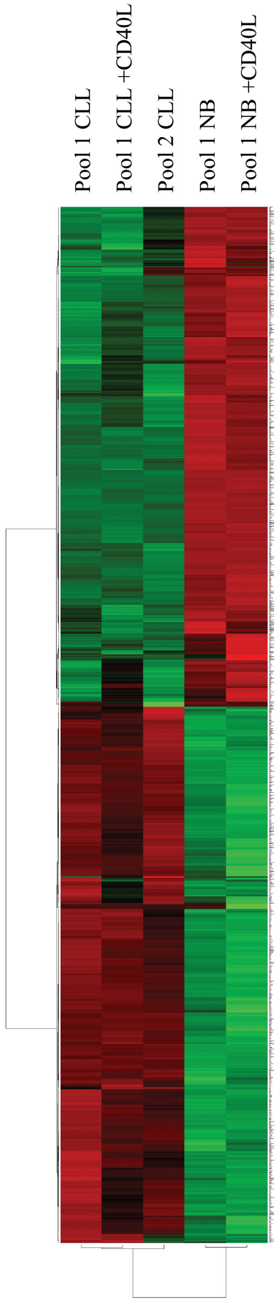

Supplementary Figure S1: Heatmap of CLL vs normal B cells lncRNA microarray. Microarray of two pools of CLL, one stimulated with CD40L, compared to one pool of normal B cells, with and without CD40L stimulation.

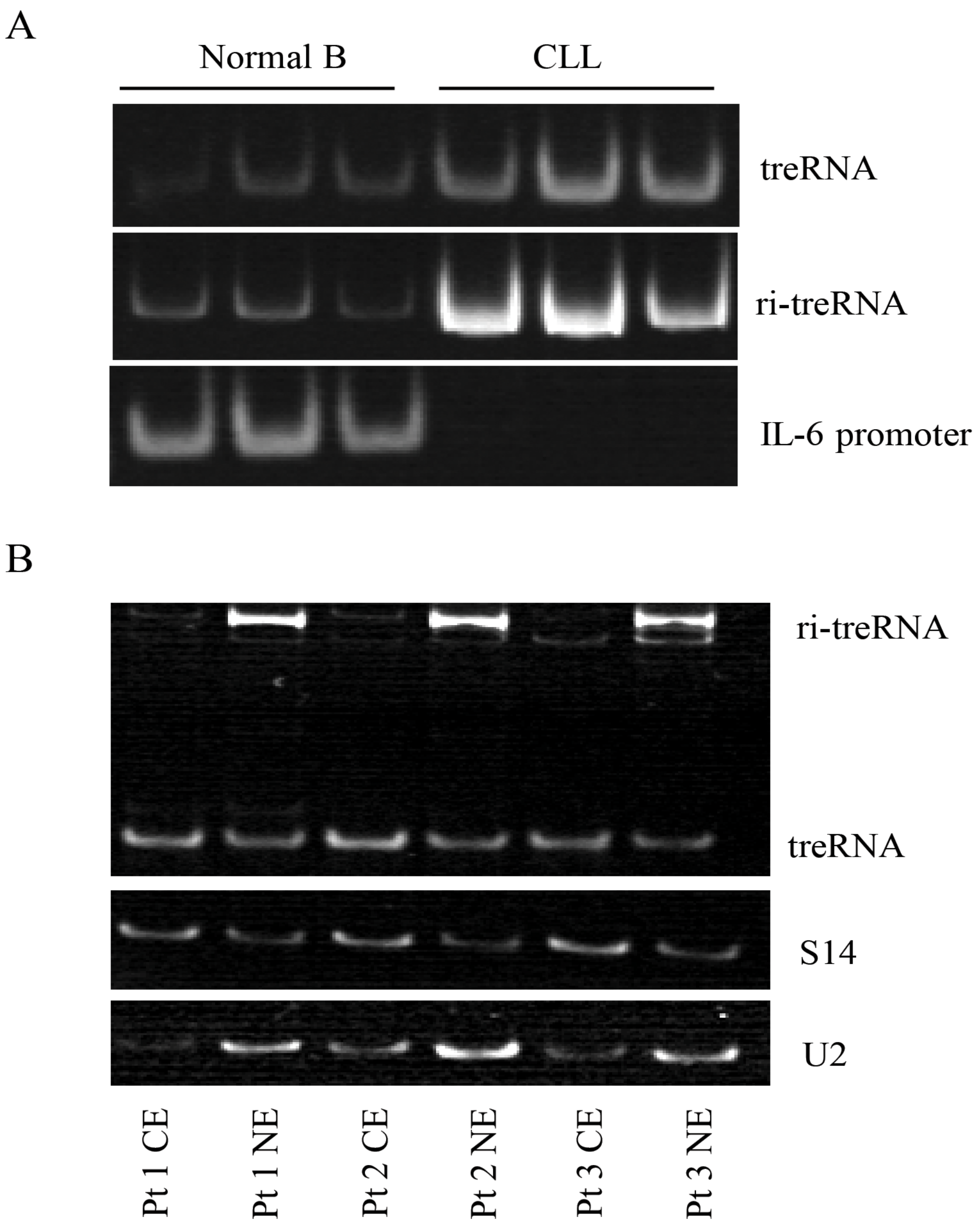

Supplementary Figure S2: CLL cells express retained intron treRNA. : (a) RT-PCR for treRNA and ri-treRNA in normal B samples and primary CLL samples. PCR for the IL-6 promoter was performed as a control to detect genomic DNA contamination. (b) : RT-PCR for treRNA, U2 (nuclear enrichment control), and S14 (cytoplasmic enrichment control) as previously described by Gummireddy et al., 2013 in nuclear (NE) and cytoplasmic (CE) fractions in primary CLL patient (pt) samples (n=3).

A

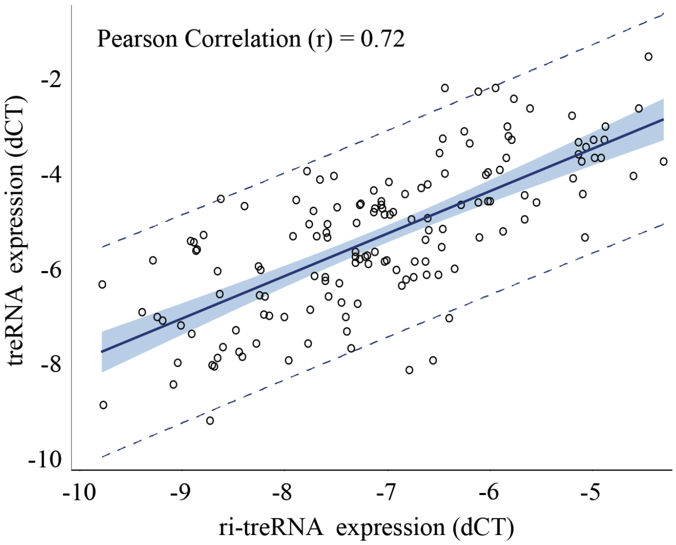

B

Variable	Low ri-treRNA (n = 73)	High ri-treRNA (n = 74)	P-value
Treatment Arm, Num. (%)			
Fludarabine (F)	34 (47)	41 (55)	0.32
F+Cyclophosphamide (C)	39 (53)	33 (45)	
Median Age, yrs. (Range)	60 (33-83)	61 (40-78)	0.22
Female, Num. (%)	18 (25)	24 (32)	0.36
Rai Stage II/III/IV, Num. (%)	55 (75)	52 (70)	0.58
Zap70 Methylation, Num. (%)			
Low	32 (70)	45 (79)	0.36
High (≥20%)	14 (30)	12 (21)	
Unknown	27	17	
IGHV, Num. (%)			
Mutated	27 (44)	18 (27)	0.07
Unmutated (≥98%)	35 (56)	48 (73)	
Unknown	11	8	
Classification			
del(17p)	5 (7)	8 (11)	
del(11q)	9 (12)	10 (14)	
+12	22 (30)	15 (21)	0.36
del(6q)	1 (1)	4 (6)	
Normal	15 (21)	9 (13)	
del(13q)	21 (29)	25 (35)	
Unknown	0	3	
del(17p)	5 (8)	8 (12)	
del(11q)	9 (15)	10 (15)	
+12/Notch Mutated	8 (13)	7 (11)	0.59
IGHV Unmutated	20 (32)	27 (41)	
Other	20 (32)	14 (21)	
Unknown	11	8	

Supplementary Figure S3: ri-treRNA expression correlates with treRNA expression but is not clinically significant. (a) Correlation between treRNA and treRNA retained intron expression measured by qRT-PCR in the ECOG-2997 patient samples. (b) Associations between ri-treRNA expression (high vs. low based on the median) and demographic and molecular variables. Associations were tested using the Wilcoxon rank sum and Fisher's exact tests. Abbreviations: yrs, years; num, number; del, deletion.

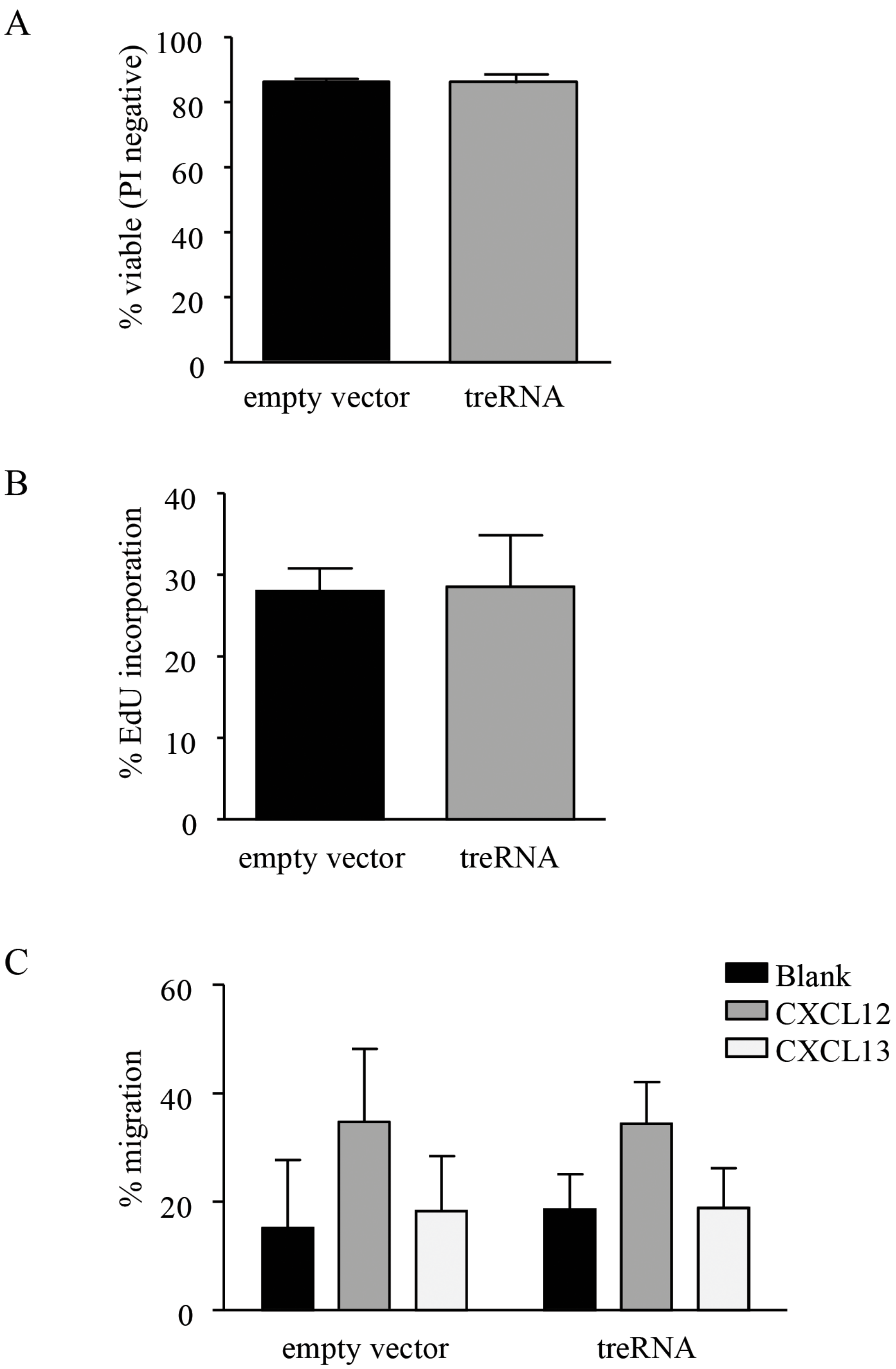

Supplementary Figure S4: TreRNA expression in OSU-CLL does not alter viability, proliferation, or migration. (a) Viability of OSU-CLL empty vector and OSU-CLL treRNA by PI staining. (b) Proliferation of OSU-CLL empty vector and OSU-CLL treRNA by EdU incorporation. (c) Transwell migration towards media or chemokine (CXCL12 or CXCL13) by OSU-CLL empty vector and OSU-CLL treRNA. Graphs display mean with standard deviations.

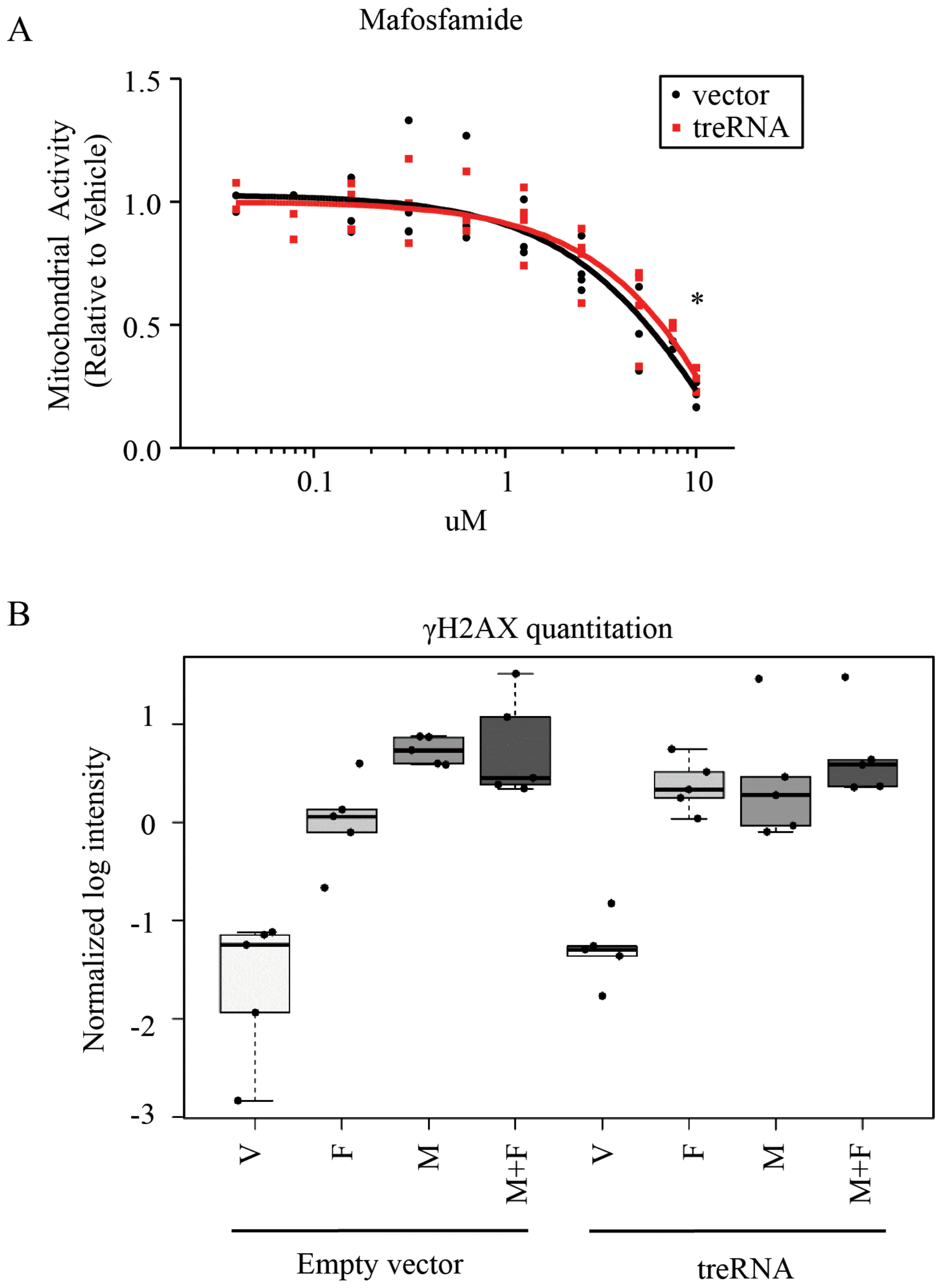

Supplementary Figure S5: Viability and γH2AX quantitation following treatment with fludarabine and mafosfamide in OSU-CLL treRNA and empty vector. (a) Viability of OSU-CLL empty vector and OSU-CLL treRNA drugged with mafosfamide for 48 hours. Viability assessed by MTS. *, p=0.001. (b) γH2AX western quantitation. Differences were not significant.

Supplementary Table S1: Primers used for gene expression studies for lncRNA in CLL.

Sybr green primers

lncRNA	Forward	Reverse complement
ENST00000503154	CCTGGGACTTCACAACACCAGCCG	CCACACTCTCTTTGGCCACTCCAGG
ENST00000456588	GGCACAGGCTGTACAGGAAGCG	GTCATGTAAGACGTGCCTGCTCTCC
ENST00000413901	GCAGCGTCCAGAGGCTGG	GGCAGCGAGGATGCTGAACC
ENST00000423967	CCACCCACCGCGTATACCTCTG	CTGGCACATCTTACCGGTTTCTGCG
treRNA	CGTGGCCGATTTGAGAGAGTGAGAC	CCAGGTCTGGGCAAAGAGAGGC
AK126772	GCTGGAGTGGCCTTGTCCTCATTC	GCCAGTCTAGGTCTACCTGAGCCTC
AK000998	GGGTTCACGCCATTCTCCTGGATGAC	GGGTTCAGGCACTGACCATGTGTTCC
lncREL1.1	TTGTAATCCCAGCACTTCAGG	AAAGAACTACGGCCAGACAC

TaqMan primers

lncRNA	Forward	Reverse	Probe
treRNA-CRC	CGTGGCCGATTTGAGAGAGTGA GACC	CCAGGTCTGGGCAAAGAGAG GC	GCTGTAGCCCTGGCAACCTC CACTCCGCCTG
treRNA-ECOG	GGTGGTTTTACGTGGCCGATTT GAG	CCAGGTCTGGGCAAAGAGAG GC	GCTGTAGCCCTGGCAACCTC CACTCCGCCTG
ri-treRNA	GTGGCCAAAAGGGGACCGAAG	CCAGGTCTGGGCAAAGAGAG GC	GCTGTAGCCCTGGCAACCTC CACTCCGCCTG
ENST000004139 01	GCAGCGTCCAGAGGCTGG	GGCAGCGAGGATGCTGAACC	GGATGCTGCTGGCAGGTGCA CCACACTGAGTTGC
